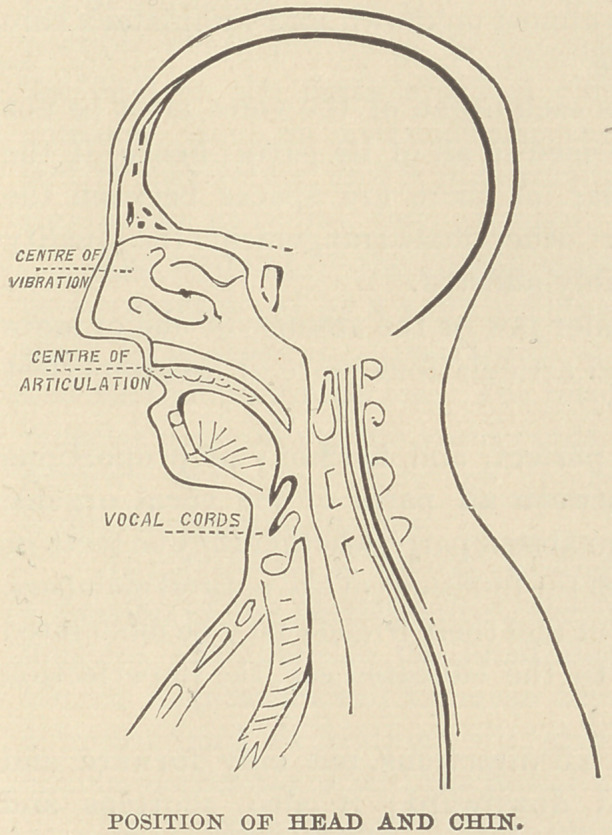# The Relation of the Teeth and Palate to Vocalism

**Published:** 1888-11

**Authors:** Thomas Fillebrown

**Affiliations:** Portland, Me.


### Independent Practitioner.

Vol. IX. November, 1888. No. 11.

Note.—No paper published or to be published in another journal will be accepted for this department. All papers must be in the hands of the Editor before the first day of the month preceding that in which they are expected to appear. Extra copies will be furnished to each contributor of an accepted original article, and reprints, in pamphlet form, may be had at the cost of the paper, press-work and binding, if ordered when the manuscript is forwarded. The Editor and Publishers are not responsible for the opinions expressed by contributors. The journal is issued promptly, on the first day of each month.

### Original Communications.

### THE RELATION OF THE TEETH AND PALATE TO VOCALISM.

BY THOMAS FILLEBROWN, M. D., D. M. D., PORTLAND, ME.

Read before the Union Meeting of the Connecticut Valley and Massachusetts Societies, Boston, Mass., July 10, 1888.

The relation of dentists to the oral cavity is such as to demand of them a thorough understanding of all that pertains to its functions, as well as its anatomy and pathology. Vocalization and articulation are among its most important functions, and worthy the thoughtful consideration of this body.

Having had considerable personal experience in vocal culture, favorable and unfavorable, and also in the forming and adjusting of obturators for cleft palate, I have been led to study the subject more or less thoroughly, and I find my conclusions so radically different from the teachings of dental text-books, that I feel constrained to offer them for your consideration.

The action of the soft palate has, perhaps, greater influence upon the tone of the voice than any other organ. I have consulted many treatises on both singing and speaking, and nearly every writer has, according to my observations, entirely misconceived the action of the velum. Drs. Flint and Lennox Brown are the more notable exceptions. It affords me satisfaction to observe that by personal consultation I find that *teachers* of singing and speaking are, in many cases, giving much better instruction than is written in the books.

Few writers, indeed, have made personal examinations on the subject, but have been content to take for granted the general ideas of others. Not a single work on oratory that I have been able to find gives any definite idea at all of the action of the organs of the human vocal apparatus and in them no attempt is made to define, describe or explain the action of the soft palate. Some physiologists have described its action, as observed by them, but in wrongly educated subjects.

Dr. Kingsley says: * "Pure vocal sounds can be made by the resonance of the buccal cavity alone. Let any other cavity communicate with it, and the purity of the vowel sounds is destroyed. If there be any escape of breath or sound, however small, behind the curtain of the palate, the vowels will be nasalised."

Dr. Carl Seiler states: † "The cavities of the nasopharynx and nose are separated from the direct influence of the vibrations of the vocal cords by the adaptation of the soft palate to the pharyngeal wall."

Dr. Seiler conceives the head cavities to be a reinforcing power, but thinks they are set in vibration through the walls of the palate, and not through an opening behind the velum.

This is entirely at variance with facts, as verified by my own experience and observation. The true office of the soft palate is not to close but to modify the opening into the nares, and thus attune the resonant cavities to the pitch and timbre of the note given by the vocal cords, throat and pharynx. A sound confined to the throat and mouth is harsh, weak, and without penetrating

* Oral Deformities.

† American System of Dentistry, Vol. III.

power; but aided by the reinforcing vibrations of the nasal and head cavities, the voice becomes soft, strong and far-reaching, and agreeable to the ear of the listener.

To understand how the palate or teeth affect vocalism, we must understand how the best tone is produced. This we can get by studying the organs themselves and their action. The organs which give forth the human voice constitute a musical instrument of great range and power. Every truly musical instrument has in it three elements—a power, a vibrator and a resonator. The violin has the bow for a power, the string for a vibrator, and a hollow body with its contained air for a resonator. The French horn has the lungs of the player for a power, the lips for a vibrator, and the gradually enlarging tube, terminating in the flaring bell shape, to produce the quality and resonance.

In all of these instruments, the quality and power of the tone depends upon the presence of these three parts, and their perfection of construction and proper relation as regards each other as to size and position, and upon the perfect use of each part.

A split sounding-board spoils the piano; a "cracked fiddle" is the synonym for everything disagreeable; and the indented bell destroys the lovely tone of the French horn.

The human vocal instrument has these three elements, and each element variable according to the will or feeling of the player. This constitutes a modifying power, which gives a variety of quality known in no other instrument, and makes it the wonder and admiration of mankind. To these is added another element—organs for articulation.

In this human instrument:

1. The lungs give the power.
2. The vocal cords are the vibrator.
3. The nasal and head cavities are the resonator.
4. The mouth and lips are the articulator.

The modification of these parts, produced by the feelings of the singer or speaker, give qualities of tone expressive of any emotion a person may feel, as pain or pleasure, joy or grief, courage or fear.

The quality and power of resonance is well illustrated by a tuning-fork, which, if set in vibration, can, unaided, be heard but a little distance, and only faintly; but if rested upon a table or plate of glass, or, better, upon the edge of the bridge of this violin, it will set up a series of vibrations of the same pitch and character, which are distinctly heard throughout this large hall. A column of air, contained in a cylinder or pipe of the size and length to reproduce the note, or a bottle with a neck the right size, will produce the same effect when the vibratory fork is held before the opening; but if the opening be stopped up, the vibrations can be only very imperfectly and faintly reproduced.

The walls and contained air of the head cavities, which consist of the mastoid and ethmoid cells, the antra, vomer, turbinated bones and frontal sinuses, present a vibratory surface of scarcely less than fifty square inches, and contain from twelve to twenty cubic inches of air, and constitute a resonator of wonderful power; but if they be shut off from the vibratory cords by closing of the velum against the posterior wall of the pharynx, their resonating power is lost, and the tone goes out undeveloped. The tuning-fork was not heard, but the vibrations of the resonant violin upon what it rested were loud and prolonged, and filled the hall. The vibrations of the vocal cords alone are insignificant. It is the vibrations of the resonant apparatus of the human instrument which give pleasure to the ear, and are sonorus and far reaching.

The nasal tone so much dreaded by vocal teachers, and the "Yankee voice" is not produced by an open palate, and the vibrations extending to the nasal passages, but by obstruction principally of the outer nasal passages by contraction of the alæ of the nose. If the nostrils be contracted by muscular actions or by outward pressure, the nasal twang will be pronounced; but if the nostrils be fully opened a full clear tone is given out. If while giving the prolonged sound of *ng* the exterior opening of the nose be alternately compressed and distended the difference in the sound will be very marked as to nasal quality. The genuine "Yankee tone" seems to be dependent also upon a contraction of the posterior nares and elevation of the dorsum of the tongue; but the pure nasal quality is produced as above described.

That the velum is drawn forward allowing a free passage into the posterior nares during the vowel sounds, I have had proven by observations. Prof. Harrison Allen, of Philadelphia, kindly gave his attention to the matter and made examinations for the purpose and found this to be the case. Dr. I. E. Kimball, of Portland, also verified the conditions, and Lennox Brown makes the same statement.

Singers cannot obtain the best quality of voice except in this way, and as speaking is only modified singing the same rule holds good for the formal speaker as for the singer. Because the singing voice is so much better understood, I have analyzed its productions to illustrate the formations and delivery of the speaking voice.

Singing is a formal continuous tone unbroken between the words. Speaking is broken between the words and syllables. Singing is confined to some particular pitch and changes from one pitch to another by regular intervals.

Speaking is unrestrained by such limits and varies without relation to pitch or interval. Yet the accomplished speaker uses very largely a definite pitch and musical tone.

The singing and speaking tones are produced by the vocal organs in the same way and in precisely the same focus with the same resonance and the same articulation is used.

A great deal is said and written about a "pure tone;" but writers do not describe it, and it is meaningless in itself.

We are told to speak and sing natural, for the natural tone is correct. This is also indefinite. What is a natural tone? It is natural for a child to imitate the first sound it hears; it may be the French nasal, the German gutteral or the American open-tone. In either case the child imitates and for it this becomes the natural tone.

To be natural is the hardest lesson to learn, and it is only the result of severe and prolonged discipline. Untrained naturalness is the perfection of awkwardness.

The involuntary functions of organic life are the only ones naturally performed correctly. Nature's method of circulation, swallowing and breathing can be depended upon, and the initial cry of the infant when ushered into the world has the *true ring*, which is recognized throughout the house. But unless established in their action by imitation and discipline, their functions will soon be corrupted by false examples.

Fig. 1.

Fig. 1 shows the position the palate and tongue should assume while giving the sounds of vowels and dipthongs—a, e, i, o ; ōo, oi, oy, ou.

The essential qualities of a tone are now recognised to be softness and resonance, the last making it far-reaching and effective. Power and volume are the product of increased resonance and largeness. Resonance is increased by the more perfect focusing of the vibrations. Largeness is improved by a general expansion of thecavities of the throat, mouth and nose, especially by depression of the tongue. To properly form and deliver a tone all the organs involved should be correctly trained and well used.

Correct breathing is very essential, and this is universally conceded to be the abdominal breathing. The lower part of the thorax is enlarged laterally, and the abdomen is enlarged both laterally and anteriorly by the depression of the diaphragm.

The shoulders should *never* be raised a particle, but should remain as fixed as were Demosthenes under the points of the swords hung above him.

Expel the breath by contraction of the abdominal muscles ; and in proportion as they are trained and strengthened will the possible force and intensity of the tone increase. The weakness of many singers is the result of weak breathing. Observe a sleeping infant; it will afford a perfect example of abdominal breathing, and no one could have a suspicion of sex from any difference in the function. In my judgment all the peculiarities of female breathing are the result of customs practiced in after life.

Fig. 2.

Fig. 2 is the profile of an accomplished vocalist and shows correct breathing. It is worthy of notice how much more the breathing capacity can be lessened than increased from the state of rest.

1 Position of diaphragm at rest.
5 " " " during full inspiration.
6 " " " " " expiration.
3 " " Chest and abdomen at rest.
4 " " " " " full inspiration.
2 " " " " " " expiration.

The larynx should rest in the position it takes during a yawning inspiration ; any bobbing around or up and down is detrimental to the quality of the tone and injurious to the organ.

The head should be inclined a *little* forward, the chin down, and the under jaw drawn back, the tongue should lie as low as possible in the mouth, and the mouth and pharynx made large.

This will throw the velum forward and open a free passage into the nares.

The principal centre of vibrations is the middle of the nose. The tone should seem to be made in the nose and head, and the vibrations can be plainly felt by placing the finger lightly on the thin bones of the nose or upon the top of the head.

POSITION OF HEAD AND CHIN.

All good singers produce their upper notes in this way; but many take the lower notes differently. I am quite fully convinced that the more nearly the voice is focused, as here described on low tones as well as high, the better the tone will be, and only in this way can the best results of which a voice is capable be obtained.

The center of articulation is apparently through the necks of the upper teeth and lip.

If these rules are observed the voice will not be disturbed by articulation, and the speech will seem to be entirely independent of the tone as the articulation of the solo singer is independent of the organ tone which surrounds her, though she sets all the air in vibration by speaking the notes.

Many theories are held as to the registers of the voice.

Some claim one, some three, others six. While one at least finds as many registers as there are notes in the compass of the voice.

Register means as I understand it a condition of the vocal organs as to position, focus or tension, of one or more parts, which changes when passing from one register to another.

My own studies lead me to the belief there is but one register, or rather no such thing, further than it applies to the compass of the voice. Such as head, middle and chest registers, are artificial divisions made by education, and to my mind a false education. Of one thing I feel sure, that if a singer or speaker will focus and deliver the tones throughout the compass of the voice, as described in this paper the questions of register need never be raised, and the difficulties of "blending the registers" will never be found.

Vocal organs used as thus described, will scarcely feel fatigue, and hoarseness will be to them almost unknown, and "minister's sore throat" an unheard of trouble.

To obtain the best results each organ of the voice must be not only well trained, but well formed in all of its parts; hence, if the teeth are mal-formed, irregular, or there are spaces between the anterior teeth, or they suffer other mal-arrangement, the quality of the voice will be disagreeably affected.

A prominent upper or under jaw or the absence of one or more teeth render vocalizations and articulations more or less imperfect and peculiar.

The palate must also be perfect, and harmony of proportions and relations must exist between all parts of the vocal organs. Artificial substitutes for lost or absent parts, whether they be teeth or palate, can never fully perform the functions of the natural members. The expectations of the patient and their friends may be moderated so far as to be fulfilled; but to the educated ear, the imperfection will be apparent.

The soft palate moves in all directions, not only forward and backward, but upward and downward; it also shortens and lengthens. As yet no obturator has been constructed that can compass more than the two first movements, consequently it cannot perfectly supply the absent part.

Dr. Kingsley's flat soft rubber velum more nearly fulfills the conditions required than any other yet invented, and if the material was not so perishable, it would be all that could be reasonably desired. The ball obturator hung in the throat is unphilosophical and un-physiological. It fills up the passage to the nares and obstructs the entrance to the resonator of the voice. Thus doing precisely what it is desirable to educate the natural velum not to do.

Hard rubber is cleanly and durable, and is the best material for this purpose.

An obturator which has served me best is one made of hard rubber nearly flat, curved to correspond to the form of the natural velum long enough to reach back against the anterior tubercle of the atlas and attached to a plate by a hinge or otherwise so as to move freely back and forth with the edges rounded, and so formed that the muscles of the split velum will just close forward of it and carry it back against the posterior wall of the pharynx during swallowing or speaking. The sizes and form of the velum at the upper portion where the hinge is attached, should just fit the notch of the cleft so the parts will just close around it tight when they contract. Such an instrument serves very nearly the purpose of the soft rubber velum, and is in harmony with the philosophy of voice production as to-day demonstrated.

The training of the larynx must be negative. The position is easily determined by a yawning inhalation. The effort of the mind must be to leave it unrestrained by the action of the supporting and surrounding muscles. The pitch is determined by the interior muscles controlling the vocal cords. Contraction of the muscles exterior to the larynx is one great cause of the throaty tone so common and so injurious.

Any exercise, as lifting, rowing or dumb-bell, which requires a fixation of the breath, will strengthen the abdominal and thoracic muscles and increase the breathing power. Full, deep and prolonged inspiration will increase the breathing capacity. Slow inspiration and expiration will give control of the muscles, and enable one to use at will the power and capacity acquired.

Many other points crowd upon our attention in connection with this, but the limits of the hour will not permit any attempt to discuss them at the present time.

## Figures and Tables

**Fig. 1. f1:**
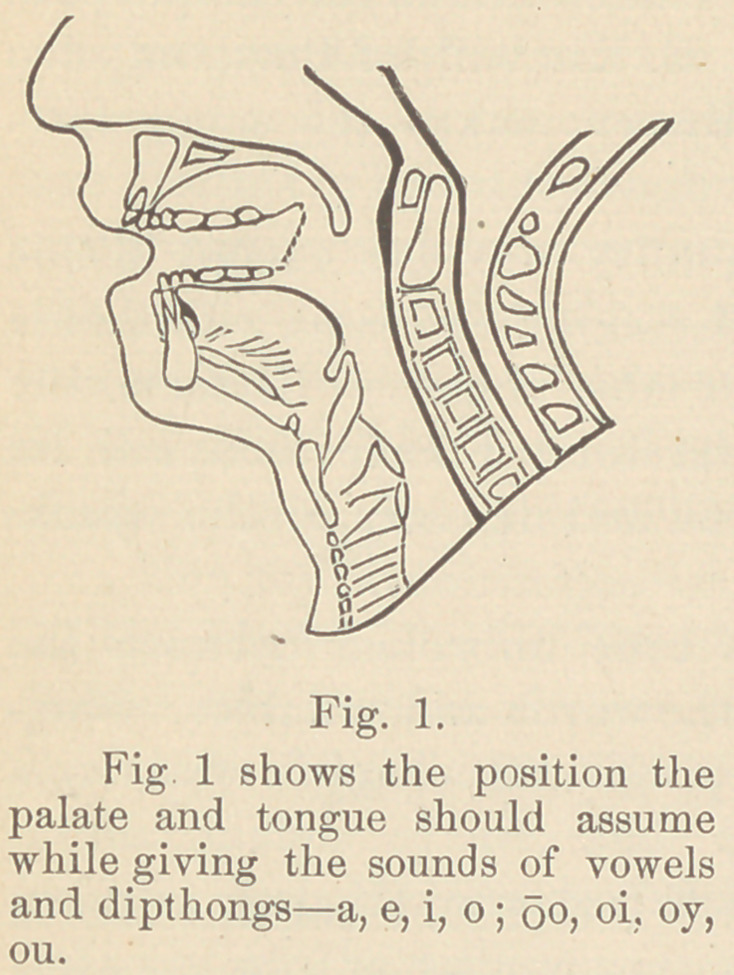


**Fig. 2. f2:**
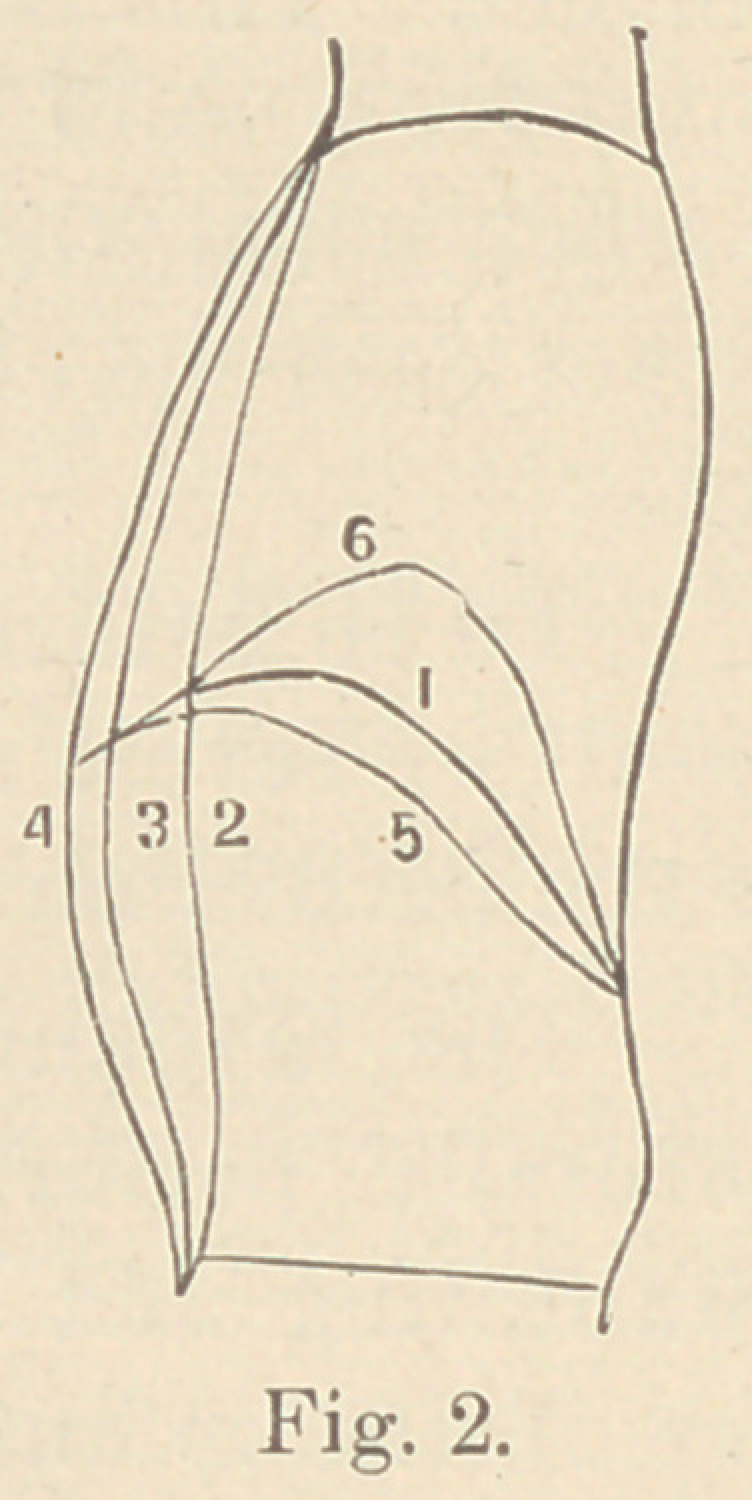


**Figure f3:**